# Prevention of disease progression in a patient with a gastric cancer-re-recurrence. Outcome after intravenous treatment with the novel antineoplastic agent taurolidine. Report of a case

**DOI:** 10.1186/1477-7819-4-34

**Published:** 2006-06-24

**Authors:** Chris Braumann, Goetz Winkler, Patrick Rogalla, Charalambos Menenakos, Christoph A Jacobi

**Affiliations:** 1 Department of General, Visceral, Vascular and Thoracic Surgery, Medical Faculty Charité, Humboldt University, Berlin, Germany; 2 Division of Molecular Biology, Medical Faculty Charité, Humboldt University, Berlin, Germany; 3 Department of Radiology, Medical Faculty Charité, Humboldt University, Berlin, Germany

## Abstract

**Background:**

Taurolidine (TRD) is a novel agent with multimodal antineoplastic effects. We present the case of a tumor remission after intravenous administration of taurolidine in a patient with gastric cancer re-recurrence.

**Case presentation:**

A 58 years old male patient suffering from a gastric adenocarcinoma was submitted to partial gastrectomy and partial liver resection (pT2, pN1, pM1_L _(liver segment 2), N0, V0). 24 months later a local recurrence was diagnosed and the patient was reoperated. Postoperatively the patient underwent a palliative chemotherapy with eloxatin, FU, and leucovorin. A subsequent CT-revealed a liver metastasis and a recurrence adjacent to the hepatic artery. After successful radiofrequency ablation of the liver metastasis the patient was intravenously treated with 2% taurolidine. The patient endured the therapy well and no toxicity was observed. CT-scans revealed a stable disease without a tumor progression or metastatic spread. After 39 cycles the patient was submitted to left nephrectomy due to primary urothelial carcinoma and died 2 days later due to myocardial infarction. Postmortem histology of the esophageal-jejunal anastomosis and liver revealed complete remission of the known metastasized gastric adenocarcinoma.

**Conclusion:**

The intravenous treatment with 2% taurolidine led to a histological remission of the tumor growth without any toxicity for the patient.

## Background

Surgical resection remains the only treatment modality offering the possibility of cure for patients with gastric cancer. Nevertheless, the extent of resection required for potentially curative operations remains controversial. Opposite to japanese studies (D3 and D4 lymph node dissection) [1], different trials in Europe and USA showed significantly lower morbidity and mortality rates with no survival difference when a less radical lymph node dissection of perigastric- (D1) or regional lymph nodes outside the perigastric area (D2) was performed [2-4]. So far, adjuvant chemotherapy in gastric cancer has failed to improve survival (e.g. 5-fluorouracil (5-FU) plus high dose methotrexate plus doxorubicin (FAMTX), etoposid plus cisplatin (EAP), etoposide plus leucovorin plus 5-FU (ELF), and epirubicin plus cisplatin plus 5-FU (ECF) [5,6]. In a locally advanced gastric cancer or in case of relapse the prognosis is very poor. Therefore, new chemotherapeutic agents are tested in order to improve survival and the quality of life.

The antineoplastic substance taurolidine was found to suppress intraperitoneal gastrointestinal tumor growth after laparotomy and laparoscopy in animals [6,7]. A single intravenous injection of 0,5% taurolidine did not affect tumor growth in rats [8,9]. Unpublished data showed a reduction of advanced intraperitoneal neoplasm after an intravenous long-term treatment (unpublished). Taurolidine induces apoptosis and inhibited tumor growth in various cells lines in vitro [10-13]. The agent was found to decrease the production of Tumor Necrosis Factor alpha (TNFα) and Interleukin-1β (IL-1β) in peritoneal macrophages [14] as well as the TNFα and the VEGF secretion by gastrointestinal neoplasms [15].

Based on these data, a male patient with a gastric cancer re-relapse was palliatively treated with this agent, undergoing 42 cycles of a 7-days'-intravenously treatment. The effects on relevant blood parameters, tumor markers, side effects, and tumor size were analyzed.

## Case presentation

A 58-year-old man (83 kilo gram, kg) presented to our Surgical Department because of a gastric cancer eight years ago. The past medical history was significant for higher PSA levels for 10 years without clinical relevance, an acute myocardial infarction which was treated with coronary arteries' stenting and an arterial occlusive disease (Fontaine IIb). The patient was submitted to surgery and a subtotal gastrectomy with a gastrojejunostomy and a partial liver resection were performed (pT2, pN1, pM1_L _(liver segment 2), N0, V0). No adjuvant chemotherapy was carried out.

During the follow-up a local recurrence has been detected in the abdominal CT-scan 24 months after surgery. The patient was then submitted to a total gastrectomy with Roux en Y esophagojejunostomy. A palliative chemotherapy (eloxatin, 5-fluoruracil, leukovorin) was performed for 10 months but no remission was observed. Twelve months later the CT-scan revealed a stable tumor disease despite chemotherapy adjacent to the hepatic artery. Then the patient underwent a palliative intravenous therapy with 2% taurolidine for seven days (per months) with a daily dose of 300 mg per kg body weight. After twelve cycles the CT-scan revealed a tumor mass reduction and the therapy was terminated. Three months later the patient experienced abdominal discomfort. Diagnostics showed one liver metastasis in segment 8 which was successfully treated with radiofrequency ablation (Figure 2). The patient was treated with taurolidine monthly ever since. After the chemotherapy no. 32 an additional primary urothelial carcinoma in two different sites of the urinary tract (left kidney, urinary bladder) The lesion in the bladder was localy excized (pTa, G2) while the kidney lesion was put under observation by the urologists. After treatment with a total of 39 cycles with taurolidine the patient was in a good clinical condition and showed no life quality deficit. At this time the CT-scan (Figure 3) showed no tumor progression next to the hepatic artery (stable disease). Four weeks after the completion of the treatment the patient was admitted to the urology department of our hospital in order to be submitted to surgical treatment of his urothelial carcinoma. A left nephrectomy was performed. Although there were no intraoperative problems the patient unexpectedly died of an acute myocardial infarct 48 hours after surgery.

**Figure 1:**
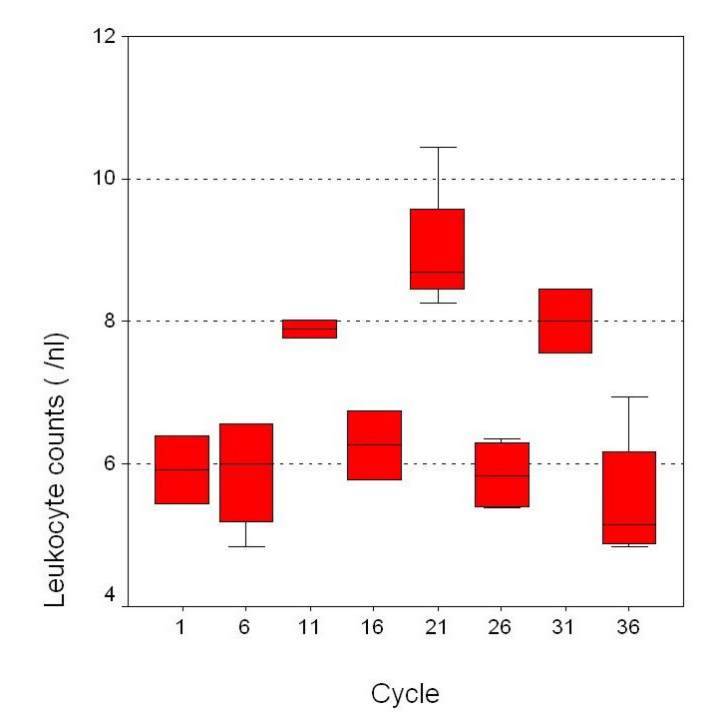
Peripheral leukocyte counts during therapy (values of every fifth cycle is shown).

**Figure 2:**
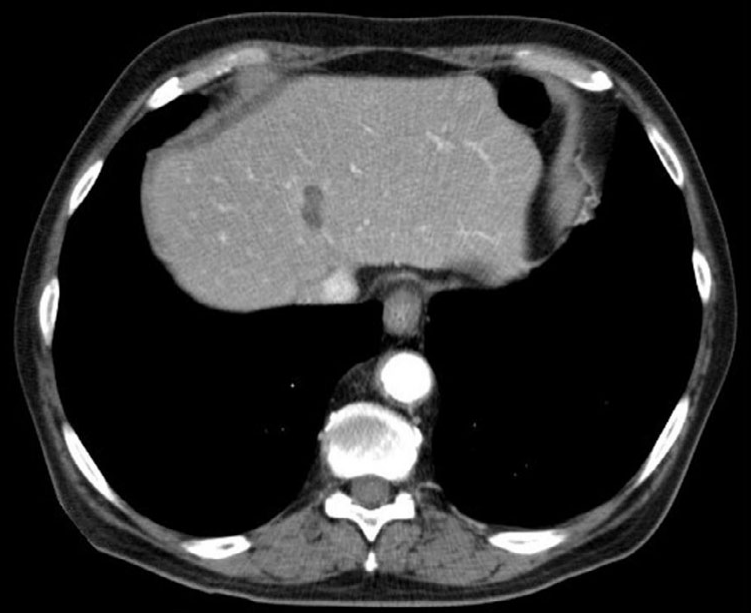
Region of thermoablation, liver segment 8.

**Figure 3:**
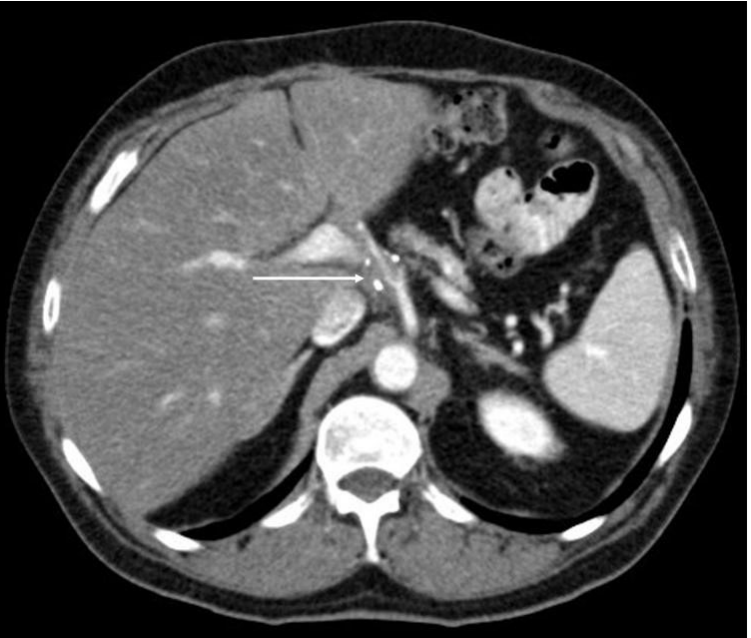
A stable disease of the gastric carcinoma surrounding the hepatic artery (arrow) could be detected after the 36 cycles chemotherapy (clips).

Post mortem histology of the esophageal-jejunal anastomosis and liver revealed surprisingly no signs of the known adenocarcinoma. We thought this finding was extremely interesting as we had actually been in front of a complete remission of his gastrointestinal tumor.

## Methods and materials

The antineoplastic agent taurolidine was intravenously applied in a 2% solution (Geistlich Pharma AG Wohlhusen, Switzerland) using a port catheter system. The monthly therapy (300 mg/kg/day according 24,9 g taurolidine/day) was given for 5 days in four hours' therapy sessions and 2 hours' intervals. Tumor markers (carcino embryonal antigen-CEA, CA 72-4) were determined at the beginning and at the end of the therapies. Blood counts and biochemistry were examined every day. Disease progression was radiologically followed-up with an abdominal CT-scan every two months.

Quality of life was assessed using the EORTC QLQ-C30 questionnaire [16], a 30- questionnaire score composed of multi-item and single-items scales reflecting the quality of life multidimensionally. It incorporates five functional scales (physical, role, cognitive, emotional, social), three symptom scales (fatigue, pain, nausea, vomiting) and a global health and quality-of life scale. The remaining single items evaluate additional symptoms commonly reported by cancer patients as well as the perceived financial impact of the disease and treatment.

GCP guidelines and Helsinki declaration for studies on humans were complied with. The Ethics Committee of the Charité Campus Mitte, University Hospital, Universitaetsmedizin Berlin, Germany, approved the study design.

## Results

The patient tolerated the therapy sessions well. As far as toxicity is concerned, we have to emphasize on the fact, that the administered taurolidine concentration (2%) did not impair the leukopoiesis or the patient's quality of life (Table 1 and 2). Tumor markers, conjugated bilirubine, creatinine or urea did not increase. Additionally the agent infusion is electrolytes-free no other side-effects from taurolidine treatment in the blood parameters were observed. Low toxicity is a main beneficial feature of taurolidine, which substantially differentiates the substance from traditional chemotherapeutic agents, which are quite often related to devastating toxic complications. According to standardized clinical and CT-scan criteria with consecutive comparisons of the CT scans during the taurolidine treatment, we were able to detect no progression of the tumor growth, so the disease was classified as stable. Moreover, it could be shown that after radiofrequency ablation liver tissue regenerates again. It has to be pointed out, that although consecutive CT-scans showed stable disease, the post mortem histological examination of the esophageal-jejunal anastomosis and liver surprisingly confirmed the absence of any residual disease.

**Table 1:** The intravenous taurolidine treatment did not impair the above mentioned blood parameters after 39 cycles (Wilcoxon test, p = not significant).

**Serum parameters**	**Median**	**Range**
CA 72-4 (tumor marker, U/ml)	0.6	0.3–1.8
CEA (tumor marker, μg/l)	18.8	6.7–32.4
Leukocyte (/μl)	6.4	4.8–13.0
Hemoglobin (g/l)	13.0	10.4–14.8
Hemocrit (%)	40	32–44
Thrombocyte (10^3^/μl)	225	171–321
Glucose (mg/dl)	94	54–283
Saline (mg/dl)	138	130–149
Potassium (mg/dl)	4.3	3.6–5.3
Creatinine (mg/dl)	1.1	0.74–1.75
Urea (mg/dl)	34	22–72
Protein (g/dl)	7.2	6.1–7.4
GOT (U/l)	23	7–95
GPT (U/l)	13	8–73
Total bilirubine (mg/dl)	0.3	0.1–0.6
Alkaline phosphatase (U/l)	70	56–122
Amylase (U/l)	53	44–104
Lipase (U/l)	17.5	17–18
INR	1.1	1.0–1.2

**Table 2:** Qualitiy of life score (mean) during intravenous TRD-treatment

	**Day 1**	**Day 5**
Physical	10.6	10.7
Emotional	4.1	4.0
Social	3.0	3.0
Fatigue	12.2	11.8
Pain	1.0	1.0
Dyspnea	2.1	2.2
Global quality of life	9.0	9.1

## Discussion

The incidence of gastric cancer has decreased worldwide. Despite this decrease, gastric cancer remains an important surgical topic, as much as its epidemiology is changing, thus making therapy for the disease more demanding. At this time, surgery is the only potentially curative therapy for this malignancy [2,4,17,18]. The aim of any surgical approach to gastric carcinoma should be complete resection with no residual tumor left behind at the end of the operation (R0 resection). Nevertheless, radical surgery after relapse is often extremely difficult – or even impossible [1] – and a standardized chemotherapy does not exist. A beneficial effect of adjuvant therapy is currently controversial. A meta analysis of 11 published trials including 2096 patients showed no significant benefit for patients who had adjuvant therapy after R0 resection [19]. Opposite to the western world, several studies clearly favoring adjuvant therapy in patients with gastric cancer have been reported from Japan, where adjuvant therapy is considered a standard modality and is initiated immediately during the postoperative period or even intraoperatively [20].

The limited therapy options with traditional chemotherapeutic substances led to intensive research for new antineoplastic agents. Taurolidine, a synthetic product derived from the aminosulfone acid taurine, consists of two aromatic rings, which are connected with a CH_2_-group; MW 284. There are several reports in the literature about the immune modulating and antineoplastic potential of this substance. Taurolidine reduced the production of TNFα (2 h contact, IC50 0.5 mM) as well as VEGF (6 h contact, IC50 1.5 mM), which is a major proangiogenic factor, might indicate a possible influence on various malignancies [15]. The agent inhibited tumor growth of many entities with poor responses to current therapeutic regiments [11,21,22]. Dose-dependent findings are supported by McCourt et al. [7] and Calabresi et al, [10]. They showed a four-fold increase of tumor cell necrosis after treatment with increasing doses in several different malignancies in vitro. The intravenous application of taurolidine had no significant side effects at a maximum concentration of 300 mg per kg per day.

We decided to treat our patient with taurolidine because he did not respond to the standard adjuvant iv chemotherapy with eloxatin, 5-fluoruracil, leukovorin. The patient developed a new local recurrence after successfully surgical treatment of his first relapse. Under these conditions taurolidine treatment appeared to be a final palliative option for our patient since traditional therapeutic modalities failed to control disease progression. In our palliative intravenous study no side effects were observed. After an intravenous therapy, leading to partial remission, the chemotherapy was terminated following the patient's wish. After a successfully radiofrequency ablation of one liver metastasis (S8) the taurolidine therapy was initiated again leading to a radiological (CT) stable disease. The patient died due to a postoperative complication after urological surgery, but post mortem histology of esophago-jejunal anastomosis and liver revealed no tumor at all. This finding was a really surprising one as it seems that taurolidine had caused a complete remission of the gastointestinal tumor (gastric cancer). This result could be only poorly expected with the conventional chemotherapy. There are literature reports that the agent might be effective in the treatment of other tumors as well. Taurolidine seems to have a multimodal efficacy on different malignancies both in human as well as in animal models. Stendel et al, noticed a partial remission of glioblastoma in two patients [23] which were treated with 20 g taurolidine per day. The substance has been found to exert a direct and selective effect on glial and neuronal brain tumor cells via presently unknown apoptotic pathways [13]. The suppression of tumor growth could be also explained by intracellular effects causing apoptosis [24] presumably by a mitochondria cytochrome c-dependent apoptotic mechanism [11], reduction of the TNFα and VEGF production on malignant tumor cells [25]. Moreover, recently the suppression of protein biosynthesis was found to lead to cell death in malignancies (IC50 approximately 1.4 mM) [15]. For example for abdominal lavage 0.5% taurolidine corresponding to approximately 16 mM are used for short time periods such as 2 hours in our clinic. The current patient received a weight-adjusted treatment (reaching up to 1.6 mM blood concentration) opposite to the glioblastoma patients (20 g each) whose therapy was successful. On the contrary, urothelial carcinoma was not affected. These findings might suggest that concentrations used exert dissimilar antineoplastic effects on different entities. The specific anti-tumor consequences have been assessed to purge tumor cells from chimeric mixtures of bone marrow resulting in a selective and complete elimination of viable cancer cells [26]. Taurolidine has been described to have immune modulating effects [24]. Therefore, peripheral leukocyte counts were analyzed to determine leukopenia which is a common side effect of other chemotherapeutic agents. In the current case leukopoesis and thrombopoesis, which occurs in the bone marrow, were not impaired. This fact should be considered as a major advantage of this agent compared to the devastating cytotoxic effects of conventional chemotherapeutics and it might implicate that the intravenous use might be safe even in immune suppressed patients in advanced tumor stages. Moreover, these findings will become more evident by using higher concentrations for other malignant entities. It is obvious that toxicity can not be assessed only in one patient. A new phase III clinical study about influence of taurolidine on gastrointestinal tumor recurrences in over 25 patients is currently being conducted in our Department. Results are expected with great interest, but first unpublished data show a favorable outcome as far as toxicity is concerned.

Taking the current findings together, the antineoplastic efficacy seems to be a mixture of the mentioned mechanisms. The intravenous 5-day-therapy of 2% taurolidine is safe and anti-tumorigenic in the advanced gastric cancer. On the contrary, urothelial carcinoma was not affected. A clinical phase clinical III study has been set up to evaluate the benefit of the intravenous efficacy on progressive tumor growth. The results are expected with great interest.

## Competing interests

The substance (taurolidine) which was administered during this phase III clinical study was supplied by the Geistlich Pharma AG, Wohlhusen, Switzerland.

## Authors' contributions

C. B. monitored the patient, collected and interpreted the data, and wrote the manuscript. G. W. collected the follow-up data and did the statistical analysis. P. R. was responsible for radiology diagnostics. C. M. was involved in drafting the manuscript and participated in the sequence alignment. C. A. J. contributed to the idea, designed the protocol, conceived and coordinated the study, gathered the data, and reviewed the manuscript. C. A. J. and C. B. are guarantors of the report.
